# Exosomes from human colorectal cancer induce a tumor-like behavior in colonic mesenchymal stromal cells

**DOI:** 10.18632/oncotarget.10574

**Published:** 2016-07-13

**Authors:** Luana Lugini, Mauro Valtieri, Cristina Federici, Serena Cecchetti, Stefania Meschini, Maria Condello, Michele Signore, Stefano Fais

**Affiliations:** ^1^ Department of Therapeutic Research and Medicine Evaluation, Istituto Superiore di Sanità, Rome, Italy; ^2^ Department of Ematology, Oncology and Molecular Medicine, Istituto Superiore di Sanità, Rome, Italy; ^3^ Department of Cell Biology and Neurosciences, Istituto Superiore di Sanità, Rome, Italy; ^4^ Department of Technology and Health, Istituto Superiore di Sanità, Rome, Italy

**Keywords:** colorectal cancer, exosomes, mesenchymal stromal cells, vacuolar H+-ATPase, CEA

## Abstract

**Background:**

Cancer cells, including colorectal cancer ones (CRC), release high amounts of nanovesicles (exosomes), delivering biochemical messages for paracrine or systemic crosstalk. Mesenchymal stromal cells (MSCs) have been shown to play contradicting roles in tumor progression.

**Results:**

CRC exosomes induce in cMSCs: i) atypical morphology, higher proliferation, migration and invasion; ii) formation of spheroids; iii) an acidic extracellular environment associated with iv) a plasma membrane redistribution of vacuolar H+-ATPase and increased expression of CEA. Colon cancer derived MSCs, which were isolated from tumor masses, produce umbilicated spheroids, a future frequently observed in the inner core of rapidly growing tumors and recapitulate the changes observed in normal colonic MSCs exposed to CRC exosomes.

**Materials and Methods:**

Tissue specific colonic (c)MSCs were exposed to primary or metastatic CRC exosomes and analysed by light and electron microscopy, proliferation in 2D and 3D cultures, migration and invasion assays, Western blot and confocal microscopy for vacuolar H+-ATPase expression.

**Conclusions:**

CRC exosomes are able to induce morphological and functional changes in colonic MSCs, which may favour tumor growth and its malignant progression. Our results suggest that exosomes are actively involved in cancer progression and that inhibiting tumor exosome release may represent a way to interfere with cancer.

## INTRODUCTION

Colon cancer remains a leading cause of death in the western world [1]. Colonic epithelial cells are the second fastest proliferating cells in the human body, are exposed to contaminants in food, and intensely interact with colonic microbiota. They originate from the stem cells located in a niche at the basis of the crypts. A transforming event can hit epithelial cells at any of their hierarchical stage, from stem cell to fully differentiated [2, 3].

During embryogenesis, organ development and injured tissue repair, stem cells are able to oscillate, in a highly coordinated fashion, between epithelial and mesenchymal states [4]. In the adult this function can be retrieved by epithelial cancer cells where epithelial-mesenchimal transition constitutes a recognized mechanism for the loss of tight junctions, the detachment of malignant cells from the primary mass, their movement through newly generated extracellular matrix toward the blood vessel walls, the crossing of the vessel wall and ultimately the colonization of distal tissues/organs [4].

Mesenchymal stromal cells (MSCs) from the bone marrow are CD146^+^ skeletal stem cells, able to generate bone, cartilage marrow, fat and hematopoietic support. Similar cells have been isolated as CD146+ pericytes, in virtually all solid tissues [5–7], where they influence the microenvironment. Colonic MSCs have been proposed to provide the niche function for colonic stem cells at the bottom of the crypts [7]. It has also been reported that tissue MSCs can be modified by tumor cells and this might explain the contradicting reports showing their inhibition or promotion of cancer cell growth [8–10].

MSCs and cancer cells communicate through exchange of signals often enclosed in exosomes. Exosomes are nanovesicles of 50–150 nm in size, containing different cell metabolites, produced and exported by all cell types. Exosomes can be detected in all human body fluids [11–13]. They have been recognized as an efficient communication system between cells, physiologically devoted to maintain homeostasis. In plasma of cancer patients the exosomes are released at very high levels [14–16] and contribute to the progression and immune escape of the disease [17, 18]. This has been demonstrated in melanoma, glioblastoma, prostate, lung, breast, and colorectal cancers [14, 15, 17, 19–21], wherein they act both in the short and in the long range [14–17, 22–24]. Melanoma produced exosomes have been shown to reach the bone marrow, recruit and reprogram bone marrow precursors to colonize the lung wherein they assemble the pre-metastatic niche [25]. In addition, also normal bystander cells release exosomes, affecting cancer cells [9]. Exosomes contain proteins, nucleic acids or lipids that may activate or transform normal cells not only at paracrine level, but also at systemic level [25]. Intriguingly, it has been shown that circulating tumor exosomes may transfer reporter genes into the germline [22].

In addition, extracellular microenvironment has been shown to have a key role in physiological or pathological conditions [26]. In malignancies, due to their fast and disorganized growth, cancer masses lack of an appropriate structure and vascular support, and are consequently nutrient and oxygen starved. The activation of aerobic glycolysis produces lactic acid and lowers the extracellular pH (Warburg effect) [27]. Thus, the finding of an acidic interstitial microenvironment and an alkaline intracellular pH is common in cancer masses [27, 28], wherein necrotic areas are also common. This hostile microenvironment induces further cell quiescence or stemness [29]. It interferes with extracellular matrix production, cell protrusion, motility and invasion [30]. Hypoxia and low pH may also influence the amount and the effect of exosomes released at the tumor site [31, 32].

The control of intracellular and extracellular pH is mediated by the vacuolar H+-ATPase (V-ATPase), either directly [33–35] or indirectly, through the overexpression of cancer-related genes and proteins [36]. V-ATPases are often over-expressed in cancer and positively correlate with its malignancy [33, 37, 38].

In this paper we show the ability of colorectal cancer (CRC) exosomes to directly induce an activation in cMSCs isolated from normal colonic mucosa. CRC exosomes induce in cMSCs: i) atypical morphology, i.e. microvilli, pseudopods, vesicles, and higher proliferation, migration and invasion; ii) formation of large 3D spheroids; iii) an acidic extracellular microenvironment linked to iv) a plasma membrane redistribution of vacuolar H+-ATPase. In addition, colon cancer derived MSCs, isolated from colon adenocarcinoma cell masses, fully recapitulate the changes observed in normal colonic MSCs exposed to CRC exosomes, supporting the idea that our experimental model fully resumes the MSCs modification consequent to the *in vivo* exposure to native exosomes inside the cancer mass.

## RESULTS

### Colorectal cancer cells-derived exosomes induce tumor-like morphological changes and marked growth rate increase in colonic MSCs

**Figure 1:**
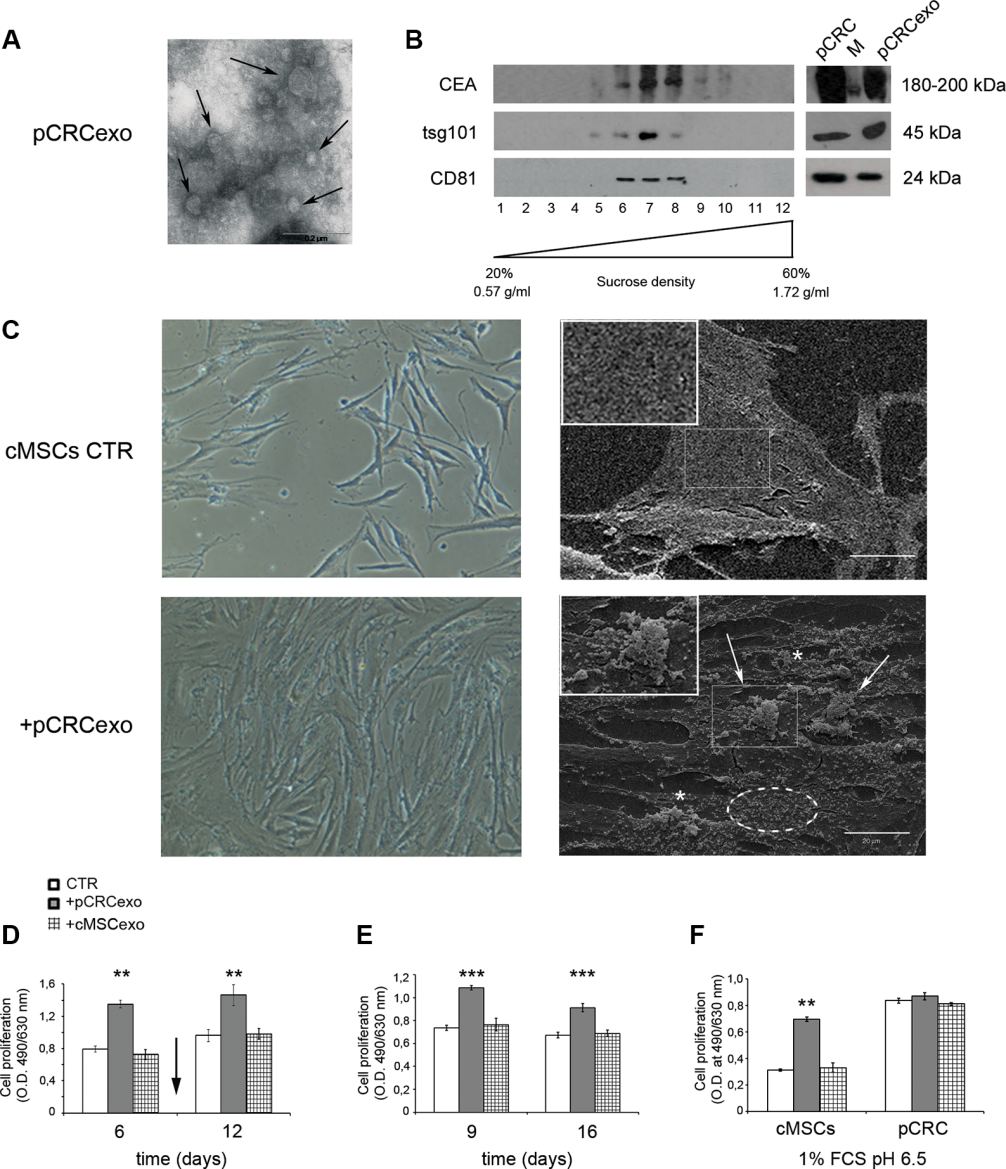
Colorectal cancer exosomes induce changes in colonic MSC morphology and growth rate (**A**) Transmission electron microscopy image of SW480 primary CRC derived exosomes (pCRCexo). Arrows indicate different size nanovesicles. Scale bar, 0.2 μM. (**B**) Western blot analysis of sucrose gradient fractions of pCRCexo blotted for the detection of carcinoembryonic antigen (CEA), tsg101 and CD81 (ubiquitous exosome markers) molecules. The density in which exosomes float corresponds to the tsg101- and CD81-positive fractions, and it is comprised between 0.90 and 1.22 g/ml. Total protein extracts of pCRC cells and their purified exosomes (pCRCexo) were loaded as control. M is the weight molecular protein marker; 1–12 correspond to the twelve fractions from sucrose density gradient. (**C**) Phase contrast microscopy (left panels) and scanning electron microscopy (SEM, right panels) images of colonic MSCs (cMSCs) treated for 6 days with pCRCexo. Arrows, asterisks and dotted circle indicate pseudopods, microvilli and vesicles respectively. 20X magnification in contrast microscopy; in SEM scale bar, 20 μM. Inserts represent a 2X magnification. Representative images of two independent experiments are reported. (**D**) Cell proliferation of cMSCs exposed to pCRCexo or cMSCs derived exosomes (cMSCexo) for 6 and 12 days; arrow indicates the exosomes re-feeding at day 9; proliferation was measured at day 6 and 12. (**E**) Cell proliferation of cMSCs incubated with pCRCexo or cMSCexo for 9 days and then replated in fresh medium without exosomes for other 7 days; proliferation was measured at day 9 and 16. (**F**) Cell proliferation of cMSCs or SW480 primary CRC (pCRC) cells incubated with pCRCexo or cMSCexo for 6 days at 1% FCS and pH 6.5 culture conditions. Results in D, E and F are expressed as optical density (mean ± SD, *n* = at least three independent sets of experiments (**p ≤ 0.005; (***p ≤ 0.001;), compared to untreated cMSCs (CTR).

The carcinoembryonic antigen (CEA) is overexpressed in several epithelial tumors and represents an important clinical marker for colorectal carcinomas [39]. CEA has been detected in extracellular vesicles from colorectal cancer patients plasma [15]. First of all we characterized exosomes derived from SW480 human primary colorectal carcinoma cell line (pCRCexo) by transmission electron microscopy (Figure 1A) and analysis in Western blot of 100 mg pCRCexo sucrose gradient centrifugation fractions (Figure 1B). In particular we searched for the ubiquitous exosome marker tsg101 and tetraspannin protein CD81 [40], floating at the expected density (ranging from 0.90 and 1.22 g/ml) of exosomes. Interestingly CEA was also expressed on pCRCexo (Figure 1B). Calregulin and nucleoporin proteins (endoplasmic reticulum and nucleus markers respectively) were not detectable in our exosome purifications (data not shown).

Colonic mesenchymal stromal MSC cells (cMSCs) were isolated from colon biopsies undergoing routine screening and not showing the presence of either inflammatory or neoplastic features; isolated cells were characterized by flow cytometry analysis as reported in Supplementary Figure S1 (details in Ref. 7).

We added pCRCexo to either cMSCs or to macrophages (ΦM, phenotypic characterization reported in Supplementary Figure S2A) to evaluate their effect. We used macrophages as control because they often are, as MSCs, detectable in tumor tissue and not primarily showing signs of abnormalities. We performed proliferation assays using different concentrations of exosomes with the same amount of cMSC cells (0,5-1-2-4-8 μg exo/1000 cells) and found that 1 μg of exosomes was the best condition for an optimal effect on cMSCs. Phase contrast microscopy showed that pCRCexo induced in cMSCs (i) a clear increase in cell density and (ii) rough morphological changes in their shape (Figure 1C, left panels). The same changes were not observed in pCRCexo/ΦM co-culture (Supplementary Figure S2B, left panels). Scanning electron microscopy showed that pCRCexo induced in cMSCs morphological changes that are considered hallmarks of malignant cells, such as atypical microvilli, pseudopods and extracellular vesicles [41] (Figure 1C, right panels, magnifications in inserts). The above changes were absent in ΦM (Supplementary Figure S2B, right panels). This result was supported by XTT cell-proliferation assay of cMSCs exposed to pCRCexo. Exosomes were added at day 1, re-feeding was performed at day 9 and proliferation was measured at day 6 and 12 showing a 50% increase in proliferation (Figure 1D). The increased proliferation was also confirmed by BrdU assay (data not shown). Furthermore, we treated cMSCs with pCRCexo for 9 days; after that cells were detached and seeded again and maintained in culture for a week without further pCRCexo addition. As shown in Figure 1E, cMSCs maintained the highest proliferative rate even after pCRCexo removal. Since malignancies often induce a catabolic microenvironment, we cultured cMSCs with pCRCexo in low serum (1% FCS) and low pH (pH 6.5) conditions. In these stringent culture conditions the increase of pCRCexo-treated cMSCs proliferation rate exceeded the 50% of that induced by exosomes released by both control cells (Figure 1F). Interestingly, the rate of colonic MSCs proliferation induced by pCRC exosomes was comparable to the baseline proliferation rate observed in primary colorectal cancer SW480 cells, with or without the addition of pCRCexo (pCRC, Figure 1F). Notably, cMSCs derived exosomes did not show to exert any detectable effect in all the analyzed cells (Figure 1D, 1E and 1F).

### CRC exosomes induce a tumor-like behaviour in colonic MSCs

**Figure 2:**
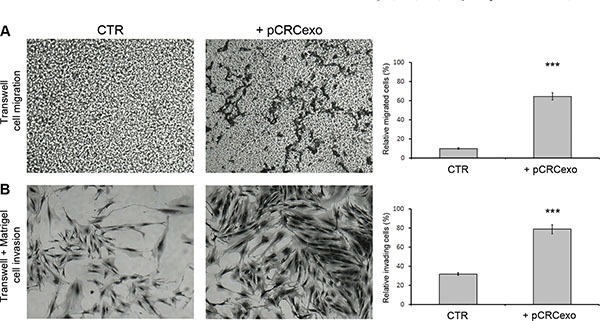
Colorectal cancer exosomes increase the migration and invasive ability of colonic MSCs (**A**) Phase contrast microscopy images of migration capability of colonic MSCs untreated (CTR) or treated for 72 hours with pCRCexo and graphic of relative migrated cell percentage. 4X magnification. (**B**) Phase contrast microscopy images of Matrigel^™^ invasion capability of MSCs treated for 72 hours with pCRCexo and graphic of relative invading cell percentage. 20X magnification. In either A and B figures, cMSCs were stained with crystal violet solution (for details see M&M) and the results were obtained analysing at least 5 fields of each sample. Data are represented as mean ± SD with *n* = at least three independent sets of experiments (***p ≤ 0.001).

cMSCs were treated with pCRCexo for 72 hours and analyzed for their migratory and invasive ability by a transwell chamber assay (for details see Material and Methods). The quantitative analysis was performed on the membranes stained with crystal violet, by estimating the percentage of cMSCs that either migrated through the transwell membrane pores or that, following migration through the pores, invaded the Matrigel™ at the bottom of the chamber. As shown in Figure 2, colonic MSCs incubated with pCRC exosomes displayed a 6-fold increase in migration (panels A) and a 2.4-fold increase in invasion (panels B). In these set of experiments we used 1 mg exo/600 cells to treat cMSCs, a larger amount compared with that used in the proliferation assay (1 mg exo/1000 cells), in order to obtain the maximal effect.

### CRC exosomes induce spheroid formation in colonic MSCs

**Figure 3:**
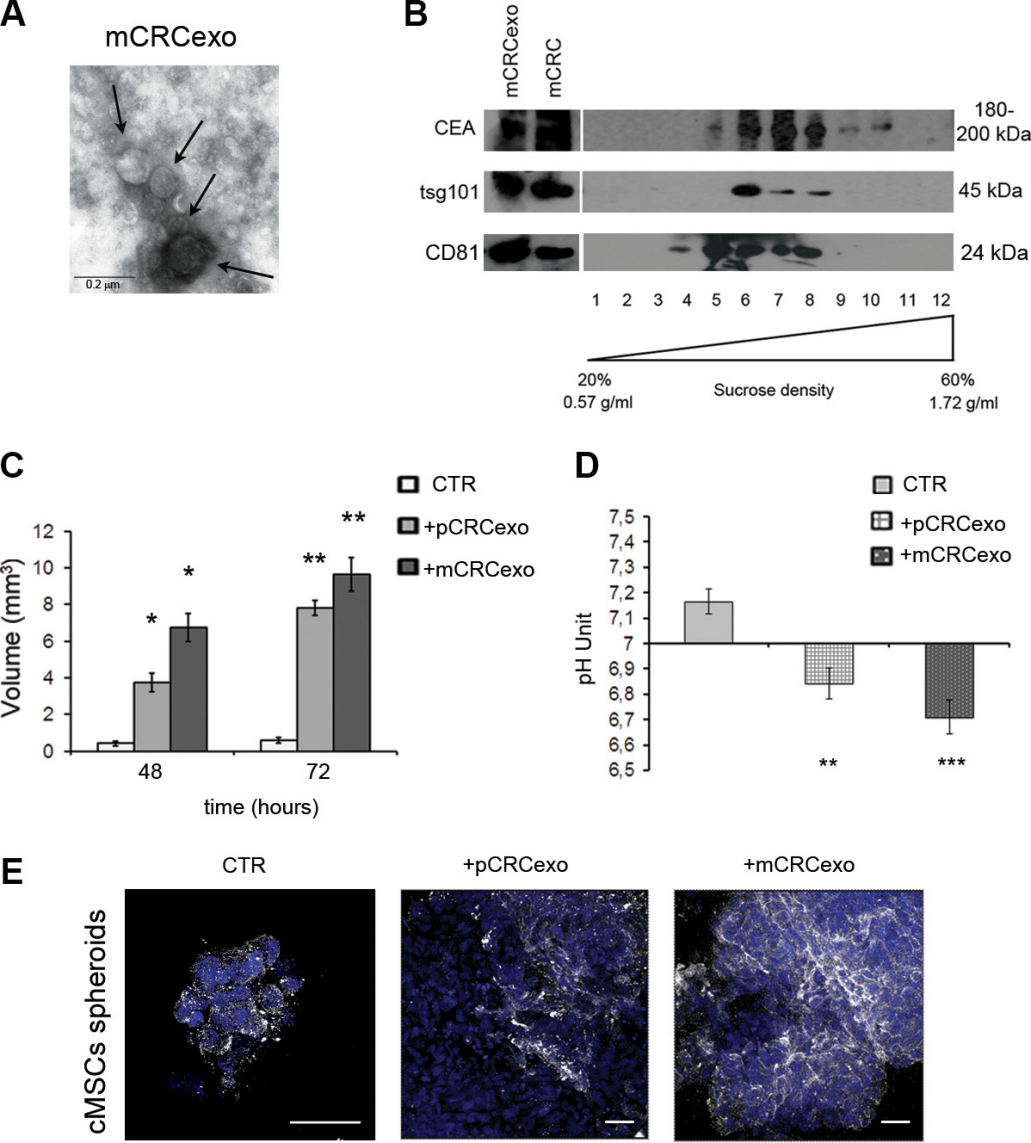
Colorectal cancer exosomes promote colonic MSC spheroids formation (**A**) Transmission electron microscopy image of SW620 metastatic CRC derived exosomes (mCRCexo). Arrows indicate different size nanovesicles. Scale bar, 0.2 μM. (**B**) Western blot analysis of sucrose gradient fractions of mCRCexo blotted with CEA, tsg101 and CD81 (ubiquitous exosome markers). The density in which CEA^+^ exosomes float, correspond to the tsg101^+^ and to the CD81^+^ fractions, and it is comprised between 0.95 and 1.25 g/ml. Total protein extracts of mCRC and mCRCexo were loaded as control. 1–12 correspond to the twelve fractions from sucrose density gradient. (**C**) Measurements of the volume of colonic MSC spheroids (CTR), formed after the pCRCexo or mCRCexo treatments at 48 and 72 h. (**D**) pH measurements of colonic MSCs spheroids supernatants, derived from pCRCexo- or mCRCexo-treated spheroids (at 72 hours) compared to supernatants of untreated ones (CTR). Statistical analysis were performed by unpaired Student *t*-test (*p ≤ 0.05; **p ≤ 0.005; ***p ≤ 0.001). (**E**) Confocal laser scanning microscopy of cMSCs spheroids incubated or not (CTR) with pCRCexo and mCRCexo and stained for V-ATPase proton pump molecule, followed by Alexa Fluor^®^-488-conjugated secondary Ab (shown in white). Nuclei are reported in blue (DAPI). Scale bar, 40 μM.

Three-dimensional cell culture systems have been recognized to better mimic the *in vivo* condition [42]. We hence performed a series of experiments in an appropriate serum free medium and polypropylene tubes to allow colonic MSC 3D spheroid formation. In this settings we additionally used exosomes derived from SW620 metastatic colorectal carcinoma cell line (mCRCexo), after their characterization by transmission electron microscopy (Figure 3A) and Western blot analysis (Figure 3B). Sucrose density gradient analysis showed that pCRCexo floated at the expected exosome density, for all the used markers (CEA, tsg101 and CD81), ranging from 0.90 and 1.22 g/ml.

Both primary and metastatic CRC exosomes start to induce large spheroids at 48 h. pCRCexo induced an increase of about 4- and 8-fold in spheroid volume and mCRCexo of about 7- and 10-fold at 48 and 72 h, respectively (Figure 3C).

To exclude that our results could be due to a difference in the uptake of the two different populations of exosomes, we performed CLSM analyses and observed that after 3 hours of incubation, both pCRCexo and mCRCexo were taken up at comparable levels by cMSCs (Supplementary Figure S3A).

Moreover, we found a significant medium pH reduction in spheroid culture medium (0.32 ± 0.06 unit with pCRCexo; 0.45 ± 0.07 unit with mCRCexo) (Figure 3D). CRC exosomes alone didn't change the pH of the cell culture medium.

The pH reduction observed in cMSCs spheroids treated with p and mCRCexo led us to investigate the expression of the vacuolar H+-ATPases (V-ATPase) protein, being a proton pump involved in the pH regulation of cell microenvironment [33–36], and shown to change its distribution following microenvironmental pH variation [34]. We analyzed by CLSM the expression of V-ATPase in cMSCs spheroids, induced by either pCRCexo and mCRCexo after 72 h of incubation. CLSM images confirmed that exosome-treated cMSCs spheroids were significantly bigger than untreated spheroids consistently with a higher level of V-ATPase expression, in particular at the plasma membrane level (Figure 3E).

### CRC exosomes affect the V-ATPase localization and CEA expression in colonic MSCs

**Figure 4:**
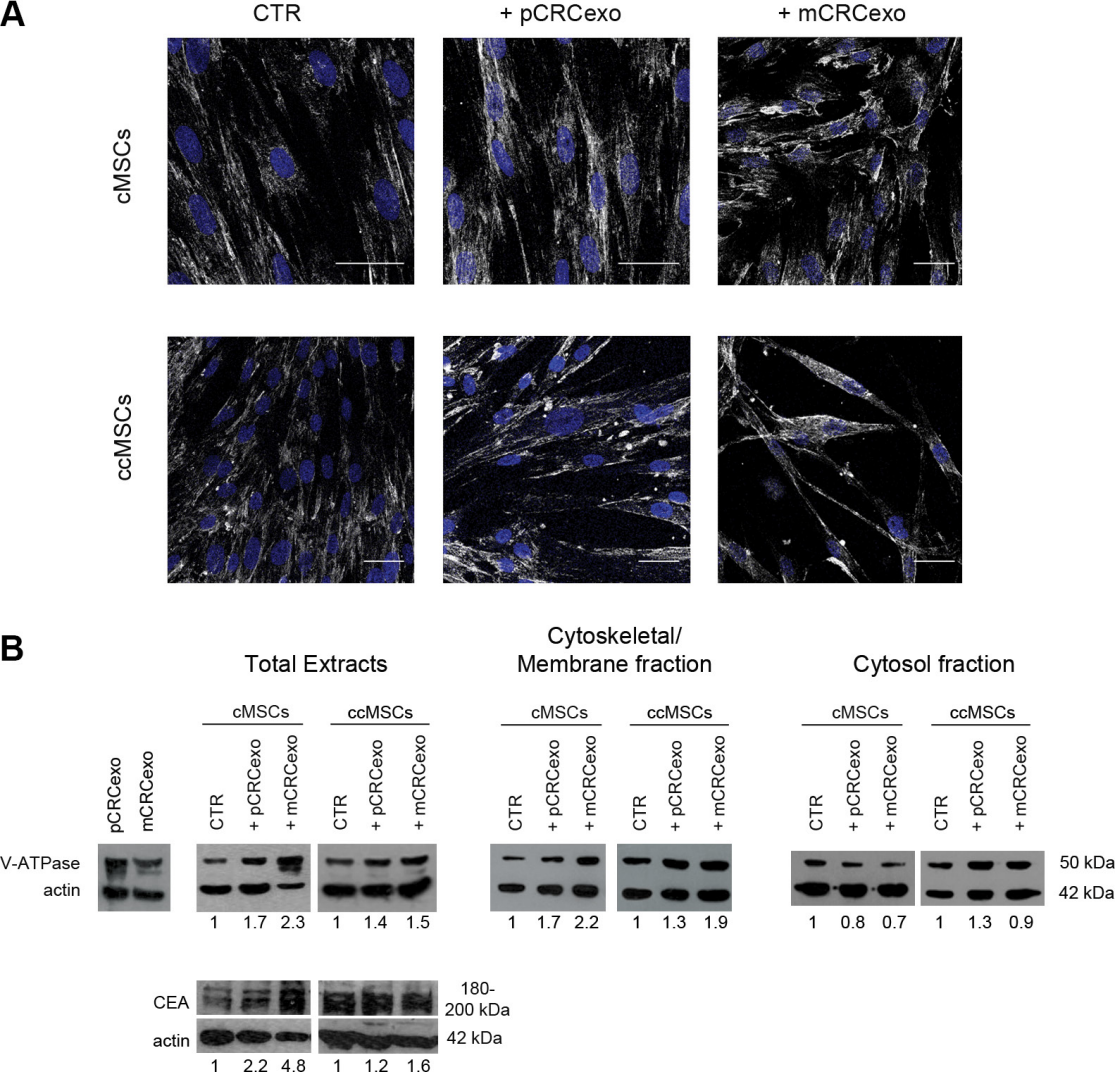
Colorectal cancer exosomes increase the expression of vacuolar H+-ATPase (V-ATPase) and CEA in colonic (c) MSCs and colon cancer (cc) MSCs (**A**) Confocal laser scanning microscopy of 5 cMSC cells optical sections Z-projection taken from the bottom to the edge of cMSCs treated with primary CRC exosomes (pCRCexo) or with metastatic CRC exosomes (mCRCexo) for 72 hours and incubated with primary anti-V-ATPase antibody, followed by Alexa Fluor^®^-488-conjugated secondary Ab (shown in white). Nuclei are reported in blue (DAPI). Scale bars, 40 μM. (**B**) Western blot analyses of V-ATPase, CEA and actin proteins, performed in total protein extracts of: pCRCexo or mCRCexo (50 μg), cMSC or ccMSC cells treated with pCRCexo or mCRCexo; Western blot analyses of V-ATPase and actin in Cytoskeletal/Membrane and Cytosol fractions; untreated cells (CTR). Results of densitometry analyses are reported as fold-increase in the expression of each molecule, related to actin loading.

We wanted to better evaluate, by CLSM and Western blot analysis, the subcellular localization of V-ATPase in colonic MSCs treated for 72 h in monolayer with either pCRCexo or mCRCexo. The confocal analysis showed that both exosome preparations induced a plasma membrane redistribution and an increase in the V-ATPase expression, that was higher in the MSCs cultures treated with mCRCexo (Figure 4A). This result was further confirmed by Western blot analyses of both protein total extracts and subcellular fractions, showing that V-ATPase expression increased in the plasma membrane/cytoskeletal fraction as compared to the cytosolic one (Figure 4B). Moreover, treatments with both primary and metastatic CRCexo increased the CEA cellular expression, as shown by the Western blot analysis of protein total extract (Figure 4B). The CEA multiple bands detectable in the Western blot analysis of the total cell extracts represented different forms of glycosylation of the molecule. Notably, Western blot analysis of exosomes preparations, showed only one form of CEA, suggesting that exosomal CEA might be exploited as a more specific tumor biomarker in the near future [43].

### Colon cancer MSCs recapitulate the functional phenotype of CRC exosome-treated colonic MSCs

To assess the behaviour of MSCs isolated and purified from colon cancers (ccMSCs) and to compare them with cMSCs derived from normal colonic mucosa, we obtained a ccMSCs line, as described in Materials and Methods. We thus performed the same analytical procedure of the previous experiments. The results, obtained by Western blot analyses, showed that control ccMSCs had a baseline high expression of both V-ATPase and CEA (respectively about 2 and 4 fold higher than untreated cMSCs). Treatment with both pCRC and mCRCexo induced i) a rather weak increase of both V-ATPase and CEA and ii) a redistribution of V-ATPase in membrane/cytoskeletal fraction (Figure 4B).

Further analysis by CLSM showed that both pCRCexo and mCRCexo were taken up by ccMSCs at the same level (Supplementary Figure S3B) and in a comparable way to that of normal cMSCs (Supplementary Figure S3A).

**Figure 5:**
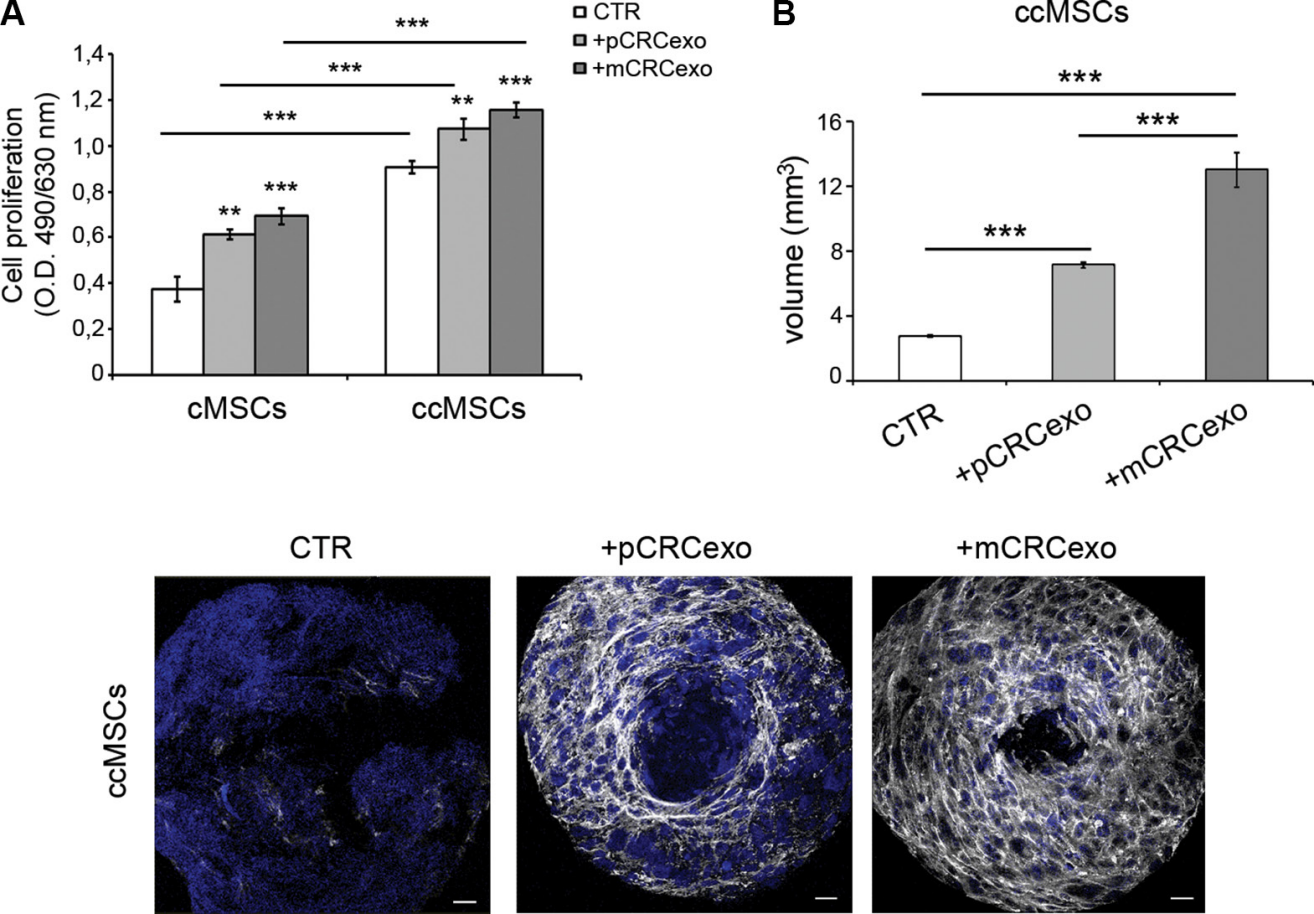
Colorectal cancer exosomes induce the umbilicated spheroids formation in ccMSCs (**A**) Proliferation rate of cMSCs spheroids compared with that of ccMSCs ones, incubated with pCRCexo or mCRCexo for 72 h; (**B**) Measurements of the volume of ccMSC spheroids formed after the pCRCexo or mCRCexo treatment at 72 h respect to untreated ccMSCs (CTR). Statistical analysis were performed by unpaired Student *t*-test, between untreated cMSCs or ccMSCs (CTR) and +pCRCexo, CTR and +mCRCexo, +pCRCexo and +mCRCexo (**p≤ 0.005; ***p < 0.001). (**C**) CLSM images of ccMSCs spheroids untreated or treated with pCRCexo or mCRCexo for 72 hours.V-ATPase protein expression was reported in white, nuclei are reported in blue (DAPI). Scale bars, 20 μM.

Actually, ccMSCs showed an increased growth rate, comparable to cMSCs exposed to either primary or metastatic CRCexo (Figure 5A). Moreover, independently from CRC exosome treatment, ccMSCs formed spheroids bigger and sooner than cMSCs (Figure 5B, compared to Figure 3C).

By confocal analysis the treatment of ccMSCs with both pCRCexo and mCRCexo produced characteristic umbilicated spheroids at 72 hours (Figure 5C), simulating the ‘necrotic center’ naturally occurring in *in vivo* tumor masses [44]. The expression of V-ATPase (in grey) resulted present and uniformly distributed only in exosome-treated ccMSCs (Figure 5C). To understand if the *in vivo* exposure of colon cancer MSCs to the native neoplastic environment was sufficient to engage the umbilicated spheroid phenotype, we isolated ccMSCs from another colon cancer, cc2MSCs. cc2MSCs, developed a large umbilicated spheroid independent of *in vitro* exosome treatment (Supplementary Figure S4), thus supporting the evidence that MSCs isolated from colon cancer have a spontaneous aptitude to form large umbilicated spheroids, in the absence of external stimuli.

## DISCUSSION

Cancer develops in the context of a microenvironment where the architects are MSCs. Their role in cancer cell growth and progression is still debated and controversial [10]. Poor prognosis colorectal cancer is associated with mesenchymal cell marker expression [45], but the mechanisms underlying this phenomenon are still obscure. Cancer-derived exosomes, including colon cancer ones, possess an active role in the disease evolution [16, 17, 25, 46]. In prostate cancer, these nanosized extracellular vesicles have shown to be able to reprogram adipose stem cells [47]. Renal cancer extracellular vesicles induce cancer promoting changes in their associated MSCs [48]. However, to date a role of exosomes released by colon cancer cells (CRC) in altering the phenotype and functional activity of normal colonic mesenchymal stromal cells (cMSCs) have not yet been reported.

Our study shows that CRC exosomes induce in cMSCs morphological and functional changes associated to the tumor-like phenotype, including atypical microvilli, pseudopods and vesicles release. These changes were exclusively detectable in cMSCs and not into macrophages. cMSCs susceptibility may depend on their stage being undifferentiated cells in contrast to terminally differentiated macrophages. Functionally CRC exosomes induce a 50% increase in cMSCs proliferation. This proliferative advantage is still detectable after a week from pCRC exosomes removal, suggesting that the exosome stimulation induces a longstanding proliferative reprogramming in cMSCs. In low serum and acidic pH condition, simulating tumor microenvironment, there is an additional 50% increase of the cMSCs proliferation rate, while untreated cMSCs underwent to proliferative arrest.

Interestingly, the proliferative rate of exosome treated cMSCs is similar to that of colorectal cancer cells. The increase in the proliferation rate is consistent with a marked increase of both cell migration (up to 6 folds) and cell invasion (up to 2.4 folds), suggesting that tumor-derived exosomes can induce a real tumor-like behaviour in cMSCs.

In this study, we used a fixed exosome concentration to treat MSCs (1 μg of exosomes on 1000 cMSCs in proliferation assay and 1/600 in migration and invasion experiments) in order to obtain the maximal effect in the different experimental settings.

Our study also shows that CRC exosomes up-regulate the expression of two cell markers in cMSCs: vacuolar H+-ATPase (V-ATPase) is considered a surrogate tumor marker, overexpressed in many metastatic cancers [49] but also a target of new anti-cancer therapies [50, 51], and CEA is a typical tumor marker [39], used worldwide for colon cancer follow up and screening. Our results have shown that V-ATPase expression impressively increases in MSCs following primary CRCexo treatment, and even more using metastatic CRCexo. Moreover, both treatments result in a clear redistribution of V-ATPase from the cytoplasm to the outer cell membrane. The overexpression of V-ATPase on the tumor cell plasma membrane is a marker of tumor malignancy and correlates with acidification of extracellular microenvironment [27]. Notably, the increase of V-ATPase in total extract of MSCs was entirely due to a redistribution of V-ATPase to the Cytoskeletal/Membrane fraction. Vacuolar H+-ATPase subcellular compartmentalization might be more relevant than its absolute amount as in Fais et al., 2007 [33]. For instance tumor cells, differently to normal cells, show a plasma membrane distribution, together to the characteristic cytoplasmic vesicles distribution [34]. This is because in tumor cells V-ATPase not only pump H^+^ within the internal vesicles, but extracellularly as well [33].

An interesting finding of our study was that tumor exosomes induced a clear increase of CEA expression in the treated cells, that was 2.2 fold increase with pCRCexo and 4.8 fold with mCRCexo. This result further supports some previous findings suggesting that tumor cell-released exosomes may have a role in the paracrine acquisition of a malignant phenotype [31]. In colon cancer derived MSCs, the baseline expression of CEA is high and it is not further increased after exposure to the CRC exosomes.

In order to mimic *in vivo* settings, we used 3D culture, that is considered a more reliable biological assay, developing a MSCs spheroid formation system [52]. The results showed that after 48 h of stimulation, primary CRCexo induced a 8-fold increase of the MSCs spheroids volume, while the metastatic CRCexo took the MSCs spheroids to10-fold the original volume. The growth of the MSCs spheroids volume was consistently associated with an acidification of the culture media, that is typical of malignant tumors [27]. In fact, a key feature of rapidly growing malignancies is the insufficient blood supply, that is often increased by the local reaction of cancer associated fibroblasts, leading to low oxygen/nutrients, high catabolites accumulation, as well [27, 28] and acidosis. Acidosis is in part due to the Warburg effect and to the overexpression and hyperfunction of proton pumps [27, 53]. These atypical microenvironment represents the major causes of the massive cell death occurring within the tumor mass [27]. Of interest, the microenvironmental tumor acidity triggers an increased tumor exosome release [31], that can be used to detoxify cells, as it has been shown with anti-tumor drugs [32]. Moreover, exosomes released in low pH have different lipid and protein make up, that appeared to favour exosome uptake by target cells [31].

We have also shown that colon cancer MSCs treated with CRC exosomes induce a quick formation of huge spheroids with a central necrosis, probably due to the very low nutrient supply caused by the rapid 3D growth. We confirmed this observation in an additional experiment performed with a colon cancer MSCs line freshly isolated from another patient biopsy, again showing a very rapid development of a huge spheroid, that was independent from the *in vitro* CRC exosome exposure. It is well known that in the large solid tumors there is a central necrosis, very often in the areas far from the vessels [44]. The observation that a central necrosis occurred centrally in the cMSC exposed to CRCexo *in vitro* suggested a comparison to what occurs within the tumor mass *in vivo*. The interaction between tumor-released exosomes and local mesenchymal stromal cells may have a key role also in the natural history of a malignant tumor.

This study shows that in colon cancer, cancer-derived exosomes induce several major changes and aberrant functions and behaviours in local mesenchymal stem cells, which may influence cancer progression [9, 26]. Tumor exosomes induce a derangement of colon-derived mesenchimal stromal cells. Colon cancer cells might thus usurp the cMSCs niche function, normally provided to crypt epithelial stem cells, to hide and maintain their own stem cell fraction that thus becomes resistant to chemotherapy, being growth arrested.

These data suggest that future anti-cancer therapies should take into account the control of exosome release by tumors, as demonstrated for anti-acidic treatments [53, 54], either *in vitro* [31] and *in vivo* [32] settings. Attempts to remove tumor exosomes from the blood stream might be greatly helpful. This may be considered a new cancer feature with a great potential to be used in the clinical follow up of colon cancer patients.

## MATERIALS AND METHODS

### Cell lines and cell culture

The isolation procedures of normal human colonic mesenchymal stromal cells (cMSCs) and of colon cancer (cc) MSCs was derived from the bone marrow MSC isolation procedure [5–7]. Briefly, cMSCs were derived from normal diagnostic colon biopsies of donors who signed an informed consent (colon surgical specimens were kindly supplied by Dr. Emanuela Pilozzi of Sant'Andrea Hospital, Rome, Italy). Few (2–3) mm of material were sufficient to develop subclonal lines of cMSCs [7]. CcMSCs and cc2MSCs were isolated from the compromised core of colon cancer biopsies. In detail ccMSCs were isolated from colon primary adenocarcinoma surgical specimens, obtained after patient signed informed consent. They were thoroughly washed in PBS with 5X antibiotic/antimycotic (A/A) solution, maintained in PBS with 5X A/A at + 4°C o/n, treated with 30–40 ml 1 μM EDTA/EGTA PBS 75′ at 20°C, vigorously shake, then counted and processed as for BM-MSCs (5). Subclonal lines of cMSCs or ccMSCS were consistently obtained from the processed samples except when sporadic bacterial contamination occurred. Colon specimens were processed as following: cell samples were treated with RosetteSep human MSC enrichment cocktail (StemCell Technologies, Vancouver, BC, Canada) composed by CD3, CD14, CD19, CD38, CD66b, glycophorin A tetrameric antibody complexes crosslinking unwanted cells with red blood cells, diluted, and centrifuged over Ficoll-Hypaque gradient for 25 min at 300 g at 20°C. Enriched cells were collected, washed, and treated with NH_4_Cl (StemCell) to remove residual red blood cells. CD34+ cells were removed by MACS column (Milteny, Bergisch Gladbach, Germany). Enriched cells were then cultured at sub-clonal density (1–10 cells/cm^2^) for 3 weeks in a-medium (Invitrogen, Carlsbad, CA), with 20% fetal calf serum (FCS; StemCell) at 37°C in 5% CO_2_/O_2_ atmosphere. MSCs immunophenotype were performed by Facs analysis, as previously described in Signore et al., 2012.

Human SW480 primary colorectal carcinoma (pCRC), human SW620 metastatic colorectal carcinoma (mCRC) cell lines (American Type Culture Collection, Manassas, VA, USA) and human healthy donor macrophages (ΦM) were cultured in RPMI 1640 (Lonza Verviers, Belgium) with 10% FCS (Lonza) at 37°C in a 5% CO_2_ environment. ΦM were obtained after separation of healthy donor peripheral blood mononuclear cell by Ficoll-Hypaque (Pharmacia, Uppsala, Sweden) density gradient and then by 46% Percoll (Biochrom KG, Berlin, Germany) density gradient of buffy coats; monocytes were left to differentiate for 2 week at 37°C in RPMI 1640 plus 20% FCS.

All cell lines were negative for mycoplasma contamination, as routinely tested by a PCR Mycoplasma detection kit (Venor GeM; Minerva Biolabs, Berlin, Germany).

### Isolation of exosomes

Exosomes were purified from culture supernatant of primary SW480, or metastatic SW620 colorectal carcinoma or colonic MSCs cell lines. The cell culture medium was subjected to differential centrifugation as previously described in standard protocol exosome preparation [40]. Briefly, cell culture medium was centrifuged for 5 min at 300 g, 20 min at 1,200 g, and 30 min at 10,000 g to remove cells and cell debris. Nanovesicles were collected by ultracentrifugation at 100,000 g for 60 min at 19°C using a Sorvall WX Ultra Series centrifuge in a F50L-2461.5 rotor (Thermo Scientific, Germany). The resulting pellet was washed in a large volume of PBS and again ultracentrifuged at 100,000 g for 60 min. Exosome pellet was resuspended in RPMI 1640 medium for MSCs treatment or dissolved in lysis buffer for analyses in Western blot or subjected to sucrose gradient floatation, as previously described [24]. Fraction density was evaluated using an Abbe' Refractometer (Carl Zeiss, Oberkochen, Germany).

### Cell proliferation assay

cMSCs were treated with exosomes derived from supernatant of primary SW480, or metastatic SW620 colorectal carcinoma cell lines (pCRCExo and mCRCexo respectively) in 1/1000 (exosome mg/MSCs cell number) ratio for different time points; proliferative rate was evaluated by the following colorimetric assays: i) cell-proliferation Kit II (XTT, Roche Molecular Biochemicals, Mannheim, Germany), results read by ELISA plate reader at 490/630 nm (Wallac VICTOR2, Turku, Finland); ii) BrdU cell proliferation Elisa Kit (Abcam) and results observed at 405 nm O.D.

For long term (16 days) and in acidic/starvation condition proliferative assays, cMSCs were cultured at 20% or 1% FCS and/or at 7.4 or 6.5 pH cell culture medium for 6 days. Acidified culture medium was obtained by adding 1N HCl.

### Migration and invasion assays

cMSCs were placed in transwell chamber with 8.0 μm pore inserts (Falcon, BD, Franklin Lakes, NJ, USA) in 96 well plates (Corning Life Sciences, Acton, MA, USA). In the invasion assays, Matrigel™ (Sigma-Aldrich) was diluted to 1 μg/mL in serum-free RPMI medium and incubated overnight at 4°C. Next day, cMSCs were seeded 24 hours, later treated with CRC-exosomes in 1/600 (exosome μg/cell number) ratio and cultured for 72 hours at 37°C and 5% CO_2_. The upper side of the insert was wiped with a wet cotton swab, while the inner side of the insert was rinsed with PBS and stained with 0.25% crystal violet solution. 595/620 nm absorbance was measured in a microplate reader (Wallac VICTOR2, Turku, Finland). Migration assay was performed as described above with the exception that inserts were not coated with Matrigel™. The values were confirmed by counting the relative number of migrated or invaded cells under a computer-assisted colour camera equipped Nikon Optiphot microscope (Nikon Corporation, Tokyo, Japan). The analysis were performed at least on 7 fields of each sample.

### Microscopy analyses

cMSCs and ccMSCs cells were incubated with or without CRC-exosomes in 1:1000 (exosome μg/MSCs cell number) ratio for 48 or 72 hours.

Images in phase contrast microscopy were acquired on live cells with a Nikon Eclipse T100 inverted microscope (Nikon Instruments Inc., Melville, NY) equipped with a LWD 20X 0.40 N.A. phase contrast objective, a Nikon DS-Fi1 color camera and the NIS-Elements F v3.0 software (Nikon Instruments Inc.).

For scanning electron microscopy examination, cMSCs, grown on coverslips, were fixed in 2.5% glutaraldehyde in 0.1 M cacodylate buffer pH 7.3, added with 2% sucrose. After post-fixation with 1% OsO_4_ in 0.1 M cacodylate buffer, cells were dehydrated through graded ethanol concentrations, critical point dried in CO_2_, (CPD 030 Balzers device, Bal-Tec, Balzers), and gold coated by sputtering (SCD04 Balzers device, Bal-Tec). The samples were then examined under a field emission gun Quanta 200 Inspect scanning electron microscope (FEI Company, Eindhoven, The Netherlands).

For confocal laser scanning microscopy (CLSM), cMSCs and ccMSCs were fixed in 3% paraformaldehyde, permeabilized by Triton X-100, and then stained with the monoclonal V-ATPase A1 antibody (H-140, Santa Cruz Biotechnology, Heidelberg, Germany), followed by Alexa Fluor^®^-488-conjugated secondary Ab. We used A1 antibody because recognizes an external and native epitope of H+VATPase. Nuclei were counterstained with DAPI (Vector Laboratories, Burlingame, CA). CLSM observations and images processing were performed with a Leica TCS SP2 (Leica, Wetzlar, Germany) [24]. CLSM images were obtained by Z-projection of 5/20 optical sections taken from the bottom to the edge of cells or spheroid-like structures, respectively. Signals from different probes were taken in sequential scan mode.

For Transmission electron microscopy, primary and metastatic colorectal carcinoma derived exosomes were fixed in 3% glutaraldehyde for 2 hours and washed with PBS two times. Exosomes were negatively stained with 2% uranyl acetate for 30 seconds, applied to a continuous carbon grid and visualized on a Philips EM208S transmission electron microscope (FEI Company, Oregon, USA).

### cMSCs and ccMSCs uptake of CRC exosomes

For uptake experiments, CLSM analysis were performed, labeling exosomes with NHS-Rhodamine (ThermoFisher Scientific, MA USA) and cMSCs/ccMSCs cells with PKH67 dye (Green Fluorescent cell linker kit; Sigma-Aldrich) as previously described (24). Image acquisition and processing were carried out using the Leica Confocal Software (Leica Mycrosystems, Wetzlar, Germany) and Adobe Photoshop software programs (Adobe Systems, San Jose, CA). Several fields were analyzed for each labeling condition, and representative results are presented.

### cMSCs and ccMSCs spheroids and pH measurement

To allow spheroid formation, 3.0 × 10^5^ colonic MSCs or colon cancer MSCs were transferred in 0,5 ml serum-free medium [55] in polypropylene 15 ml conical tubes (Falcon, BD) and incubated with or without CRC exosomes in 1:1000 (exosome mg/cell number) ratio until 72 hours at 37°C and 5% CO_2_ in continuous rotation. Then cell spheroids were transferred in chamber slides (Falcon, BD), fixed in 3% paraformaldehyde, washed and analysed by a Nikon Eclipse T100 inverted microscope (Nikon Instruments Inc.) or by CLSM. The volume of the cMSCs spheroid structures were calculated using the following formula: (4π/3)xa^2^c, where a is the equatorial radius of the spheroid, c is the distance from center to pole along the symmetry axis. The pH of cMSCs spheroids supernatant was estimated by the use of a pH 123 Microprocessor pH Meter (Hanna Instruments, Italy).

### Western blot analyses

cMSCs, ccMSCs, SW480, SW620 cells were lysed in AKT buffer (20 mM Tris-HCl pH 7.4, 150 mM NaCl, 10% glycerol, 1% NP40), while CRC-exosomes in lysis buffer (1% Triton X-100, 0.1% SDS, 0.1 M Tris HCl, pH 7.0) and processed as previously described [24]. Plasma membrane/cytoskeleton and cytosolic fractions were obtained following a previous described protocol [56]. 50 μg of total exosome proteins were loaded on SDS-PAGE, whereas 100 μg of exosome preparation were subjected to sucrose gradient flotation. Membranes were incubated with the following primary antibodies: anti-CEA (EPCEAR7, ab133633, Abcam, Cambridge, UK), anti-V-ATPase H (G-2, Santa Cruz Biotechnology), anti-V-ATPase A1 (Santa Cruz Biotechnology), anti-tsg101 (C-2; Santa Cruz Biotechnology) and anti-actin (Sigma-Aldrich, St. Louis, MO). We used A1 antibody because binds to an epitope of the denaturated protein and it works in Western blot. Then, membranes were incubated with the appropriate HRP–secondary antibody (Amersham Biosciences, Milan, Italy). Membranes were revealed by enhanced chemiluminescence (Pierce, Rockford, IL) and densitometry results were performed by the Image J software (NIH, USA).

### Statistical analysis

Data are presented as means ± SD with *n* = at least three independent sets of experiments and for triplicate wells/experiment. The statistical analysis was performed by Student's *t* test in all the reported experiments and the statistically significant differences were defined only when *p* < 0.005, using SigmaStat software.

## SUPPLEMENTARY MATERIALS FIGURES


