# Supplementary material for: Exosomes from human colorectal cancer induce a tumor-like behavior in colonic mesenchymal stromal cells
Source: Oncotarget. 2016 Jul 13;7(31):50086–98. doi: 10.18632/oncotarget.10574 (PMC5226570; doi:10.18632/oncotarget.10574)
Supplement: Supplementary file 1 [file oncotarget-07-50086-s001.pdf]

Exosomes from human colorectal cancer induce a tumor-like behavior in colonic mesenchymal stromal cells

Supplementary Materials

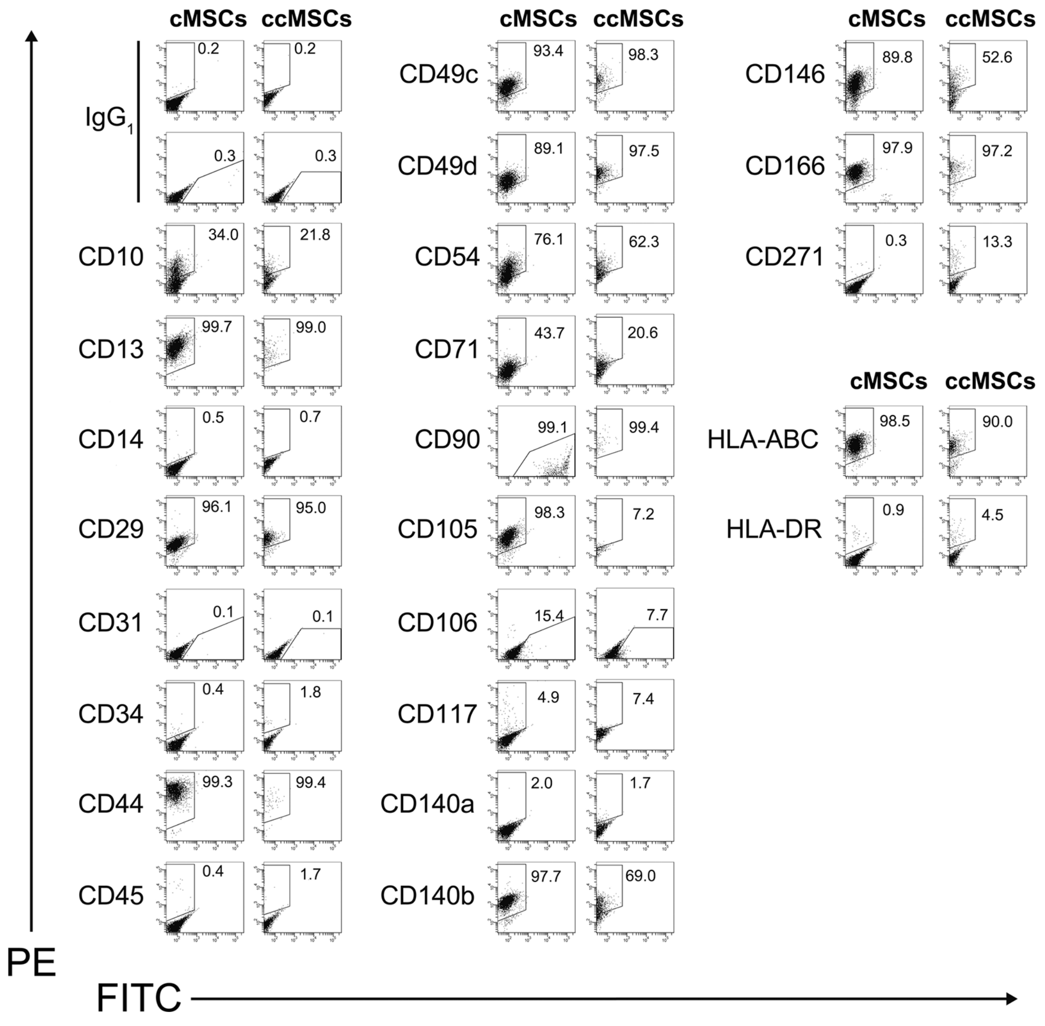

Supplementary Figure S1: Flow cytometry characterization of colonic MSCs (cMSCs) and colon cancer MSCs (ccMSCs), as in part reported in Signore et al., 2012 manuscript.

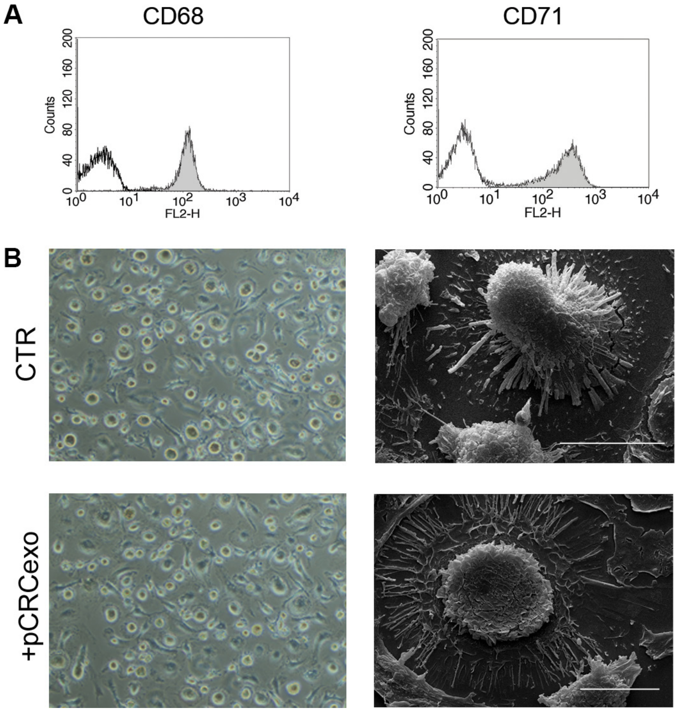

Supplementary Figure S2: (**A**) Flow cytometry characterization of macrophage cells. (**B**) Phase contrast microscopy (left panels) and scanning electron microscopy (right panels) images of macrophages (ΦM) treated for 6 days with SW480 primary CRC exosomes (pCRCexo). 20X magnification in phase contrast microscopy images; 20 µM scale bar in scanning electron microscopy images. Representative images of two independent experiments are reported.

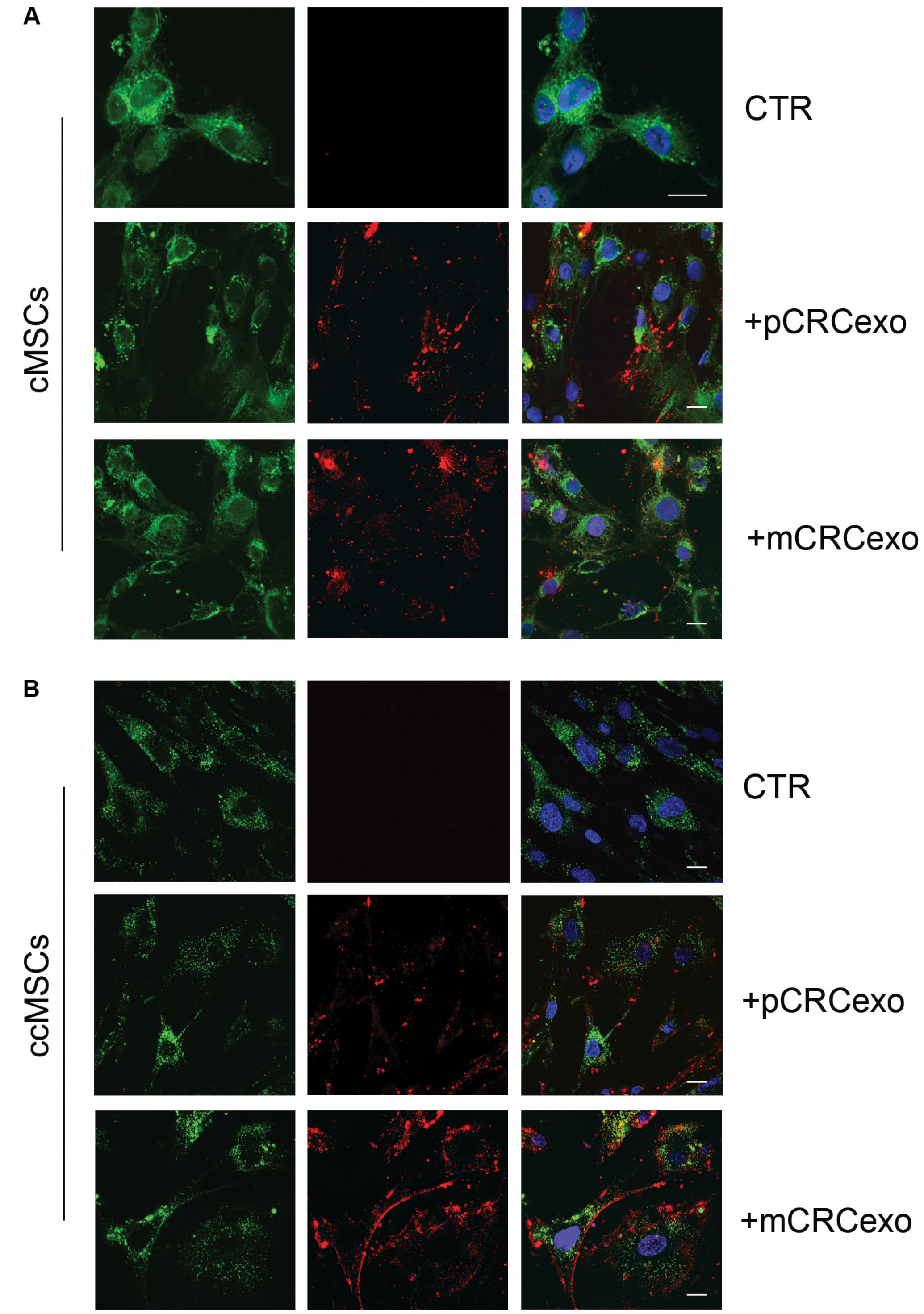

Supplementary Figure S3: Colorectal cancer exosomes are internalized by colonic and colon cancer MSCs. Confocal laser scanning microscopy of cMSCs (**A**) and ccMSCs (**B**) cells, both labeled with PKH-67 green fluorescent dye, incubated for 3 hours with pCRCexo or mCRCexo, both labeled with NHS-Rhodamine red fluorescent dye. Nuclei are reported in blue (DAPI).

cc2MSCs

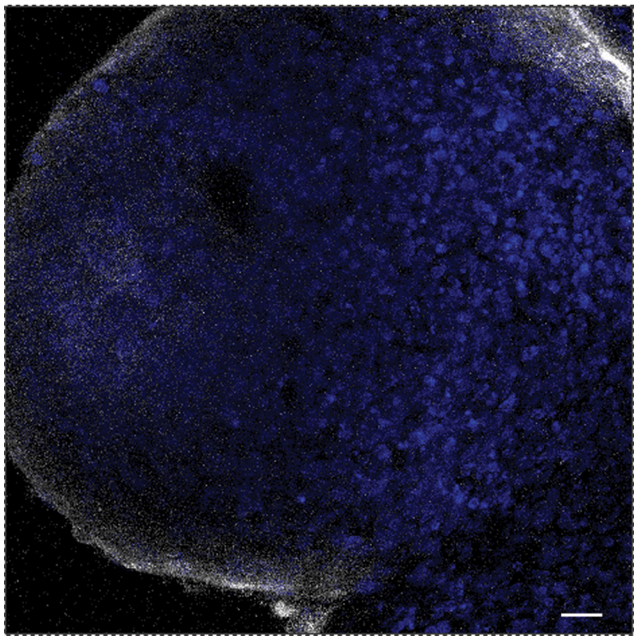

Supplementary Figure S4: Confocal laser scanning microscopy of a spheroid from a colon cancer MSCs (cc2MSCs) obtained in specific culture condition for 72 hours, as described in Materials and Methods. V-ATPase protein expression was reported in white, nuclei are reported in blue (DAPI). Scale bars, 20 μM.
